# Prognostic impact of incomplete revascularization in coronary artery bypass grafting: Association between residual SYNTAX score, magnetic resonance imaging, myocardial injury, and cardiovascular events

**DOI:** 10.1097/MD.0000000000042478

**Published:** 2025-05-30

**Authors:** Diogo Freitas Cardoso de Azevedo, Whady Hueb, Eduardo Gomes Lima, Paulo Cury Rezende, Cesar Higa Nomura, José Antonio Franchini Ramires, Roberto Kalil Filho

**Affiliations:** a Department of Clinical Cardiology, Heart Institute (InCor) University of São Paulo, São Paulo, Brazil.

**Keywords:** coronary artery bypass, coronary artery disease, SYNTAX score, troponin

## Abstract

Cardiac biomarkers are frequently released after revascularization. Incomplete revascularization may be a potential mechanism of action. This study aimed to investigate the relationship between the completeness of revascularization, quantified by the residual SYNTAX score (rSS), and myocardial injury, infarction, and cardiac events after coronary artery bypass grafting. This study included patients with stable coronary artery disease who underwent surgery. Troponin levels, cardiac magnetic resonance, and late gadolinium enhancement were assessed before and after the procedure. The baseline SYNTAX score was determined from the angiograms, and the rSS was measured based on the operative report for each patient after the procedure. Of the 136 surgical patients studied, no significant correlations were found between the rSS and median peak troponin level (*R* = 0.06, *P* = .47). The rSS was not a predictor of myocardial infarction, as assessed by late gadolinium enhancement (odds ratio [OR] 0.96, 95% confidence interval [CI] 0.86–1.03; *P* = .51) and release of troponin higher than the median (OR 1.00, 95% CI 0.92–1.08; *P* = .93). After multivariate analysis in a model including variables, only cardiopulmonary bypass time was a significant predictor of troponin peak release, which was higher than the median (OR 1.01, 95% CI 1.002–1.019; *P* = .01). During the 5-year follow-up, the incomplete revascularization group had more major cardiovascular events than the complete revascularization group (rSS = 0) (*P* log-rank = .006, adjusted hazard ratio = 11.32; *P* = .001). Myocardial injury and infarction were not significantly associated with the completeness of revascularization. However, the rSS had a prognostic impact during follow-up for cardiovascular events.

## 1. Introduction

Current interventional and surgical procedures are designed to achieve complete myocardial revascularization, as the standard approach for coronary artery disease. Patients with incomplete revascularization experience a higher rate of cardiovascular events than those with complete revascularization regardless of the myocardial revascularization strategy.^[1,2]^

The residual SYNTAX (synergy between percutaneous coronary intervention with taxus and cardiac surgery) score (rSS) score was developed to evaluate the completeness of myocardial revascularization after revascularization procedures, whether percutaneous or surgical.^[3,4]^ The calculation was performed by analyzing the description of the proposed procedure compared to the complexity of the patient’s baseline coronary anatomy using coronary angiography. The untreated lesions determine the rSS value. An rSS equal to zero therefore represents complete revascularization and is different from zero, incomplete revascularization.^[5]^

The purpose of this study was to evaluate the completeness of coronary artery bypass grafting (CABG) objectively calculated through rSS and its relationship with the release of cardiac troponin (cTn) above the 99th percentile upper reference limit (URL), myocardial injury, and type 5 periprocedural myocardial infarction, through late gadolinium enhancement (LGE) by cardiac magnetic resonance (CMR), an independent prognostic predictor for cardiovascular events,^[6,7]^ performed pre- and postoperatively, and finally, cardiovascular events.^[8]^

## 2. Materials and methods

This was a prospective and prespecified analysis of the Medicine, Angioplasty, or Surgery Study V trial,^[5]^ including essential details on the study design, protocols, and patient selection criteria, which were previously documented. The study received institutional review board approval and informed consent was obtained from all participants involved.

In summary, the trial included patients who exhibited angiographically confirmed proximal multivessel coronary stenosis >70%, as assessed visually, and a preserved left ventricular ejection fraction (>55%). Ischemia was verified using stress-testing protocols, whereas angina severity was evaluated using the Canadian Cardiovascular Society classification system. All participants were potential candidates for elective interventions, either percutaneous coronary intervention or coronary artery bypass grafting, with referrals made at the heart team’s discretion.

Key decisions regarding patient treatment were informed by a combination of clinical criteria, frailty evaluation, angiographic findings, SYNTAX score, and various imaging modalities, including CMR imaging and echocardiography. A left ventricular ejection fraction exceeding 55% was confirmed using echocardiographic assessments or CMR. Importantly, this analysis focused solely on surgical patients, whereas those who underwent prior mechanical interventions, experienced recent thromboembolic events, suffered from systemic inflammatory diseases, or had renal failure were excluded.

### 2.1. SYNTAX score calculation

The patients’ angiograms were evaluated using the SYNTAX score (SS). Each coronary lesion with a diameter ≥50% luminal obstruction in vessels with a diameter ≥1.5 mm was analyzed separately during the score calculation. The scores were summed to produce an SS, calculated using the algorithm available on the SYNTAX website (https://www.syntaxscore.com/calculator/start.htm). SS was calculated by 2 interventional cardiologists who applied the tool without knowledge of other patient information, including pre-procedure biomarker values and clinical variables. The rSS was calculated in agreement with the surgeons by comparing the diagnostic angiograms with surgical procedure reports. Thus, an rSS of 0 indicates complete revascularization, whereas an rSS of >0 indicates incomplete revascularization.

### 2.2. Myocardial revascularization

The surgical team appears to have adhered to optimal protocols for CABG procedures. Their objective was to attain complete anatomical revascularization by addressing all viable stenosed arteries and employing internal mammary conduits. The surgeries were performed by skilled surgeons proficient in both the on-pump and off-pump techniques. For myocardial protection, cold crystalloid cardioplegia was applied, and for CABG procedures performed without cardiopulmonary bypass (CPB), an octopus stabilizer from Medtronic, Inc. was used.

### 2.3. Cardiac biomarkers

Blood samples were collected to measure high-sensitivity troponin I (hs-TnI) and CK-MB levels at various time intervals during medical procedures. Specifically, samples were collected immediately before the procedure and at 6, 12, 24, 36, 48, and 72 hours after the procedure. Notably, the treating surgeon and the clinical team were blinded to the hs-TnI and CK-MB results throughout the study to ensure objectivity. hs-TnI was measured using the ADVIA Centaur immunoassay analyzer (Siemens Health Care Diagnostics), which has defined standards for sensitivity and specificity. The lower limit of detection for hs-TnI was 0.006 ng/mL, with a 99th percentile URL established at 0.04 ng/mL. The assay precision was represented by a coefficient of variation of 10% at a threshold of 0.03 ng/mL. For CK-MB, the detection limit is set at 0.18 ng/mL, with cutoff values at the 99th percentile being 3.8 ng/mL for women and 4.4 ng/mL for men. In diagnosing myocardial injury and type 5 perioperative myocardial infarction, the study adhered to the fourth universal definition of infarction, which classifies myocardial injury when cTn levels exceed the 99th percentile URL. Additionally, it outlines that CABG-related myocardial infarction is identified when cTn levels rise greater than ten times the 99th percentile URL in patients with normal baseline values. To substantiate the diagnosis, at least one of the following criteria must be met: imaging evidence indicating a new loss of viable myocardium or the emergence of a new regional wall motion abnormality suggestive of ischemia, as evaluated by CMR imaging and LGE, the recognized gold standard for assessing acute myocardial infarction.^[8]^

### 2.4. Cardiac magnetic resonance

All patients underwent CMR imaging before and after CABG during their hospital stay. A 1.5 Tesla (Philips AchievaVR) magnetic resonance scanner was used, with images acquired on 2 long axes (2 and 4 chambers) and between 8 and 10 short axes of the left ventricle. The gadolinium-based contrast agent was then injected intravenously (0.1 mmol/kg body weight). Delayed enhancement of CMR was performed using a phase-sensitive inversion recovery sequence following the administration of a contrast agent. The slice thickness at the apex was reduced to 5 mm to avoid partial-volume effects. Two experienced observers analyzed the images with the addition of a third observer when consensus was not obtained initially and without knowledge of biochemical and surgical data. New LGE areas were defined as an image intensity >2 SDs above the mean intensity in a remote region of the myocardium in the same image and quantified using the computer-aided Planimetry program CMR42 (Circle Cardiovascular Image, Calgary, Canada). To avoid mistakes, a visual assessment was also performed after a semiautomatic assessment. Moreover, pre-intervention and post-intervention scans were read side by side in all patients.

### 2.5. Patients follow-up

Patients were scheduled for periodic outpatient visits, starting 1 month after the procedure, with subsequent follow-ups every 6 months during the first year and then annually thereafter. The aim was to use optimal medical treatment to keep all patients asymptomatic. Each patient was placed on a comprehensive medical plan that included a stepped-care approach using nitrates, aspirin, beta-blockers, calcium channel blockers, and angiotensin-converting enzyme inhibitors, alone or in combination, unless contraindicated.

Additionally, statins were prescribed, and dietary recommendations included a low-fat diet tailored to individual needs. Blood pressure, lipid levels, and glucose levels were managed in accordance with established guidelines.^[9,10]^ Notably, the investigators involved in the protocol were unaware of any biochemical results related to elevated myocardial injury biomarkers or CMR reports. It is important to emphasize that all patients received similar treatment approaches, independent of changes in biomarker levels or the presence of new LGE.

### 2.6. Trial outcomes

The primary outcome of this study was the duration to the first occurrence of a composite endpoint.

1. Death.
2. Myocardial infarction is defined as a situation in which troponin levels rise above the 99th percentile, along with clinical signs or electrocardiographic evidence of ischemia.
3. Subsequent revascularization procedures.
4. Hospitalization for cardiovascular issues, specifically including:

○ Unstable angina: Characterized by an exacerbation of the angina pattern, manifesting as increased frequency, intensity, or duration of symptoms.
○ Heart failure: Identified by symptoms such as shortness of breath experienced during exertion, orthopnea, nocturnal paroxysmal dyspnea, or swelling in the lower limbs.

### 2.7. Statistical analysis

The Kolmogorov–Smirnov test was used to evaluate the distribution of continuous variables. Quantitative variables were expressed as means and SD when normal or median and interquartile ranges (IQR) when the normality test was rejected. Qualitative variables were expressed as absolute and relative frequencies. Continuous variables with normal distribution were compared using the Student *t* test, and those with non-normal distribution were compared using the Wilcoxon rank-sum test. Assessment of homogeneity between proportions was performed using the chi-square test or Fisher exact test, as appropriate. Spearman correlation analysis was applied to continuous variables, when appropriate. Multivariate logistic regression analysis was performed to evaluate the relationship between SS, rSS, biomarker elevation above the 99th percentile, median, 10 times the 99th percentile, and late enhancement in a model that included clinical, demographic, laboratory, and angiographic data. To construct the multivariable model, we first examined univariate models; variables that were at least marginally associated with the endpoint (*P* < .10) were included in a model in which stepwise selection was used for predictor selection at each step. Additional candidate variables were included in the multivariable model if there were significant treatment by predictor interactions (*P* < .05).

Event rates were estimated using the Kaplan–Meier method, and differences between groups were assessed using the log-rank test. Hazard ratios were estimated using the Cox proportional hazards analysis. Statistical significance was set at *P* < .05. These analyzes were performed using SPSS version 21.0 (SPSS Inc).

## 3. Results

**Figure 1.**
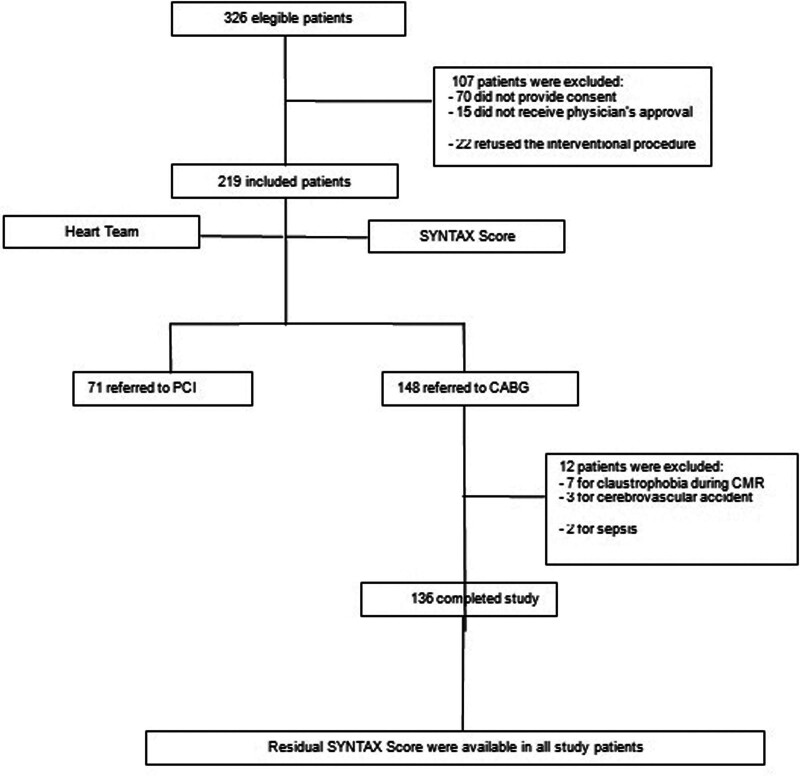
Consolidated standards of reporting trials (CONSORT) diagram of trial participants. CABG = coronary artery bypass grafting, PCI = percutaneous coronary intervention, SYNTAX = synergy between percutaneous coronary interventions with taxus, CMR = cardiac magnetic resonance.

Between March 2012 and March 2014, 326 non-randomized, prospective, consecutive patients with documented stable CAD were eligible for revascularization procedures. All patients had severe obstructive lesions in at least 2 epicardial arteries associated with angina pectoris and preserved ventricular function. A total of 148 patients were referred for CABG. Of these, 136 completed the study and were included in the primary analysis. SS and cardiac biomarkers were available for all patients included in the main analysis. SS was one of the criteria used for decision making by the local heart team. The rSS was calculated for all patients after the surgical intervention. The main causes of exclusion are shown in Figure 1.

### 3.1. SYNTAX score and residual SYNTAX score

Of the 136 surgical patients studied, 78 underwent incomplete revascularization (rSS > 0) and 58 underwent complete revascularization (rSS = 0). The mean preoperative SS was 23.3 ± 9.23 and the mean rSS after CABG was 3.36 ± 4.37; the medians of hs-TnI and CK-MB were 3.10 (IQR: 1.55–8.02 ng/dL) and 21.12 (IQR: 12.49–45.24 ng/dL), respectively. There was a difference in the rSS between patients with incomplete and complete revascularization (5.9 ± 4.3 × 0, *P* < .001), as well as in the SS value (26.2 ± 9.5 × 22.1 ± 6.9, *P* = .02). Other baseline characteristics were similar between the groups with incomplete and complete revascularization, respectively, including number of diabetic patients (43 × 27, *P* = .32), ejection fraction (58 ± 9 × 56 ± 9, *P* = .15), CK-MB peak (39.27 ± 41.72 × 31.15 ± 35.85, *P* = .09), troponin peak (8.53 ± 12.09 × 6.10 ± 9.46, *P* = .19), 3-vessel (61 × 41, *P* = .32), number of grafts (3.11 ± 0.64 and 3.22 ± 0.68, *P* = .245), LGE after CABG (24.65% × 15.51% *P* = .199), fibrosis after CABG (6.3 ± 6.9 g × 4.3 ± 5.9 g, *P* = .11) and cardiopulmonary bypass time in minutes (37.09 ± 43.81 and 34.98 ± 38.43, *P* = .77), Table 1.

**Table 1:** Demographic, laboratory, clinical, echocardiography, and angiographic characteristics of patients by residual SYNTAX score groups.

Characteristics*	Incomplete revascularization (n = 78; rSS > 0)	Complete revascularization (n = 58; rSS = 0)		*P* value
Demographic profile				
Age (yr)	63 ± 8	61 ± 10		.33
Male (%)	58 (74.3%)	36 (62.1%)		.12
Current or past smoker (%)	34 (43.5%)	28 (48.3%)		.59
Peripheral artery disease (%)	15 (19.2%)	9 (15.5%)		.57
Hypertension (%)	69 (88.4%)	47 (81.0%)		.23
Diabetes mellitus (%)	43 (55.1%)	27 (46.5%)		.32
Chronic obstructive pulmonary disease (%)	3 (3.8%)	1 (1.7%)		.47
Laboratory values				
Creatinine clearance (mL/min)	67.57 ± 16.24		71.81 ± 19.29	.17
Troponin peak (ng/dL)	8.53 (±12.09)		6.10 (±9.46)	.19
CK-MB peak (ng/dL)	39.27 (±41.72)		31.15 (±35.85)	.09
Total cholesterol† (mg/dL)	163 (128–186)		165 (146–184)	.49
LDL† (mg/dL)	93 (69–114)		93 (81–118)	.27
HDL† (mg/dL)	38 (32–44)		34 (29–41)	.06
Triglycerides† (mg/dL)	120 (90–162)		129 (84–208)	.59
C-reactive protein† (mg/dL)	3.38 (1.75–6.75)		3.29 (1.98–6.90)	.74
Angiographic and imaging findings				
LVEF (%)	58 (±9)	56 (±9)		.15
LA† (mm)	35 (33–38)	35 (31–38)		.73
LVEDD† (mL)	71 (67–77)	70 (66–78)		.74
LVESD† (mL)	34 (31–38)	38 (32–40)		.08
LGE† after CABG (%)	24.65	15.51		.19
Fibrosis after CABG† (g)	6.3 ± 6.9	4.3 ± 5.9		.11
Triple-vessel disease (%)	61 (78.2%)	41 (70.7%)		.14
SYNTAX score	26.2 ± 9.5	22.1 ± 6.9		.02
Residual SYNTAX score	5.9 ± 4.3	0		<.001
Cardiopulmonary bypass time (min)	37.09 ± 43.81	34.98 ± 38.43		.77

CABG = coronary artery bypass grafting, CK-MB = creatinine phosphokinase fraction MB, HDL = high density lipoprotein, LA = left atrial, LDL = low density lipoprotein, LGE = late gadolinium enhancement, LVEDD = left ventricular end diastolic diameter, LVEF = left ventricular ejection fraction, LVESD = left ventricular end systolic diameter, rSS = residual SYNTAX score, SYNTAX = synergy between percutaneous coronary intervention with taxus and cardiac surgery score.

* Values are expressed as means (standard deviation) or percent (number).

† Values are expressed as median (interquartile range).

**Figure 2.**
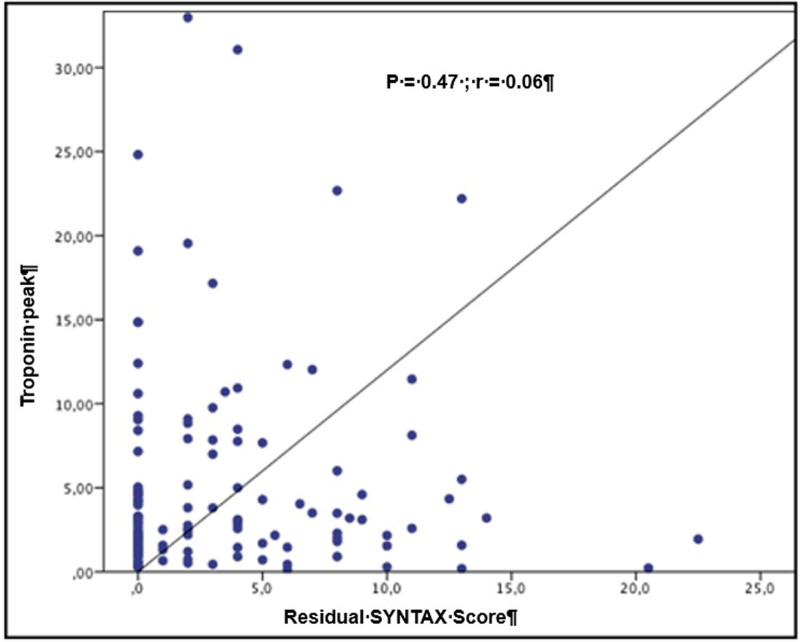
Correlation analysis between cardiac troponin peak and residual SYNTAX score.

No significant correlations were observed between rSS and median post-procedure peaks of hs-TnI (*R* = 0.06, *P* = .47) or CK-MB (*R* = 0.11, *P* = .16) levels (Fig. 2).

Twenty-seven patients had new late gadolinium enhancement after myocardial revascularization (19.8%) and elevated cTn values >10 times the 99th percentile URL. However, rSS was not a predictor of myocardial infarction, as assessed by LGE, according to the 4th universal definition of myocardial infarction (odds ratio [OR] 0.96, 95% confidence interval [CI] 0.86–1.03; *P* = .51) and for hs-TnI release higher than the median (OR 1.00, 95% CI 0.92–1.08; *P* = .93). After multivariate analysis in a model including age, sex, smoking status, diabetes, hypertension, SS, rSS, CPB time, and number of bypass grafts as variables, only CPB time was a significant independent predictor of a post-procedure hs-TnI peak release greater than the median (OR 1.01, 95% CI 1.002–1.019; *P* = .01), Table 2.

**Table 2:** Adjusted multivariate analysis for troponin release above the median coronary artery bypass grafting.

Clinical and angiographic variables	Odds ratio (95% CI) troponin peak > median	*P* value
SYNTAX score	0.98 (0.92–1.10)	.35
Residual SYNTAX score	1.08 (0.92–1.10)	.86
CPB time (for each minute)	1.01 (1.01–1.02)	.03
Number of bypass grafts	0.88 (0.40–1.58)	.68
Diabetes mellitus	1.33 (0.64–2.76)	.43
Hypertension	0.65 (0.23–1.86)	.51
Smoking status	0.63 (0.36–1.11)	.11
Age (for each year)	1.00 (0.96–1.04)	.95
Male	0.46 (0.20–1.03)	.06

CPB = cardiopulmonary bypass.

**Figure 3.**
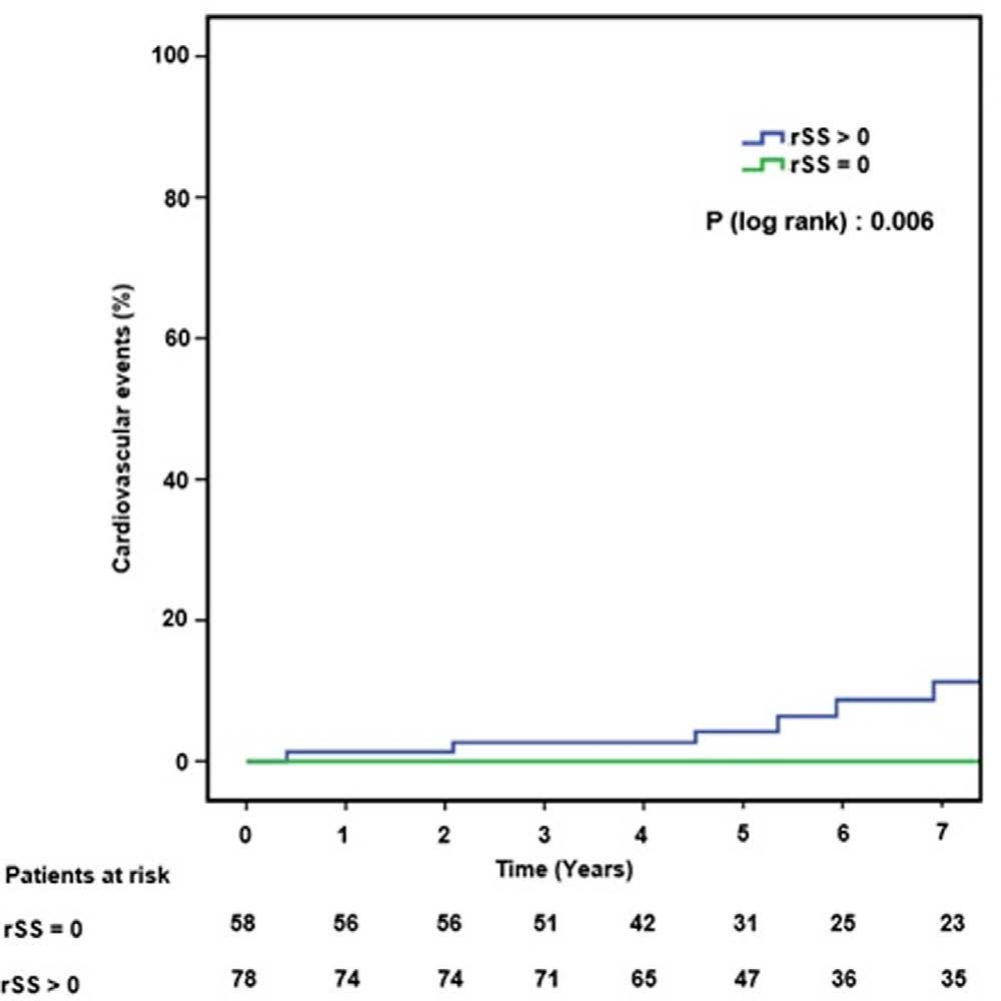
Cumulative incidence of cardiovascular events in relation to completeness of revascularization by the residual SYNTAX score. rSS = residual SYNTAX score.

During the 5-year follow-up, 11 patients in the incomplete revascularization group had major cardiovascular events compared to no patients in the complete revascularization group (rSS = 0) (*P* log-rank = .006, adjusted hazard ratio = 11.32; *P* = .001), Figure 3.

## 4. Discussion

To the best of our knowledge, this is the first study to evaluate the completeness of revascularization using the rSS and its correlation with myocardial injury and type V acute myocardial infarction after CABG using the noninvasive gold standard method for the diagnosis of acute myocardial infarction and cardiac magnetic resonance.

Residual SS was not a predictor of myocardial injury or acute infarction according to the fourth universal definition of myocardial infarction, the main reference used in the literature and was chosen in the present study.^[8]^ Both groups showed troponin elevation 10 times the URL in >90% of myocardial revascularization surgeries; however, only 27 patients showed late enhancement on cardiac magnetic resonance analysis. Therefore, in this study, rSS was not a predictor of late enhancement on CMR after uncomplicated surgery. It is important to mention that all patients in the complete and incomplete revascularization groups underwent CMR before and after revascularization procedures; 272 high-cost examinations were performed using the appropriate technique and with a defined methodology for analysis. The simple use of biomarkers for the diagnosis of type V myocardial infarction loses specificity and can lead to overdiagnosis and overtreatment, in addition to the psychological impact on patients at such vulnerable times.

Both groups showed similarities in clinical, angiographic, and echocardiographic characteristics including left ventricular ejection fraction, number of 3-vessel diseases, and CPB time. However, the values of rSS and SS were different. It is rational that patients with higher SS have a higher rate of incomplete revascularization (rSS > 0). Classically, in several studies and meta-analyses, there has always been a dichotomized approach to CABG, such as complete versus incomplete revascularization.^[1]^ Patients with incomplete revascularization have a worse prognosis during the long-term follow-up.^[9–11]^

The SYNTAX score is an angiographic assessment tool with the capacity to objectively characterize and quantify the severity and extent of coronary artery disease. It is most widely used in revascularization guidelines because of its practicality and ability to predict prognosis, including in very long-term follow-up of 10 years.^[12,13]^ The rSS, derived from SS, allows an objective definition of revascularization completeness with prior validation. Farooq et al^[3]^ reported higher mortality in the population undergoing percutaneous treatment with rSS > 8 than in those undergoing complete revascularization during 5 years of follow-up. Takahashi et al,^[4]^ when analyzing the CABG arm, did not identify any difference in cardiovascular outcomes in the SYNTAX study population because of the completeness of revascularization or rSS. Melina et al^[14]^ evaluated cardiovascular outcomes 1 year after CABG according to the rSS and suggested that the rSS may be a useful tool for risk stratification of patients undergoing the first isolated myocardial revascularization. These findings agree with our results, since combined cardiovascular outcomes were more frequent in the rSS > 0 group. The low event rate in the rSS = 0 group reflects the epidemiological characteristics of this population, which underwent uncomplicated CABG in patients with preserved ventricular function, stable coronary artery disease, and complete revascularization. Thus, it appears that the release of biomarkers and short-term injury in the absence of acute myocardial infarction is related to CPB time, operative technique, and various mechanisms of injury (incomplete myocardial protection with transient ischemia, reperfusion, systemic inflammatory state, etc) rather than to the completeness of revascularization or rSS. In contrast, the best long-term prognosis is due to the greater protection of vascular territories at risk (rSS = 0).

### 4.1. Study limitations

When interpreting the findings of a non-randomized analysis, limitations must be considered. This study was a non-randomized evaluation of the Medicine, Angioplasty, or Surgery Study V trial; thus, the results are best viewed as generating hypotheses rather than confirming them. It is worth noting that despite the robustness of the methodology of the present study, the number of patients included for CABG was limited. One key point is that the findings may not be applicable to other hs-TnI assays, as different manufacturers employ distinct reference populations to establish the 99th percentile URL, meaning these assays are not biologically equivalent. Consequently, the implications of this study should be restricted to a defined subset of patients with comparable characteristics, specifically those with preserved left ventricular ejection fraction in the context of chronic coronary syndrome following uncomplicated revascularization procedures. These results should not be generalized to other clinical scenarios. Moreover, the analysis did not examine the short-term patency of grafts following CABG, which represents another limitation. Other cardiac biomarkers, such as natriuretic peptide, were not measured because of the exclusive analysis of patients with preserved left ventricular function. However, this biomarker, which is a clear indicator of myocardial strain stress, would provide interesting data in conjunction with the analysis of myocardial injury markers, such as hs-TnI and CK-MB, supported by current guidelines after myocardial revascularization procedures.^[8]^

## 5. Conclusion

The completeness of revascularization, assessed using rSS, was not associated with the occurrence of myocardial injury or myocardial infarction, with CPB time being the only independent predictor of troponin release after CABG. However, complete revascularization, objectively quantified by zero rSS, had a prognostic impact on long-term follow-up.

## Author contributions

**Conceptualization:** Whady Hueb, Diogo Freitas Cardoso de Azevedo, Roberto Kalil Filho.

**Data curation:** Whady Hueb, Diogo Freitas Cardoso de Azevedo, Paulo Cury Rezende.

**Formal analysis:** Diogo Freitas Cardoso de Azevedo, Eduardo Gomes Lima.

**Funding acquisition:** Whady Hueb.

**Investigation:** Whady Hueb, Diogo Freitas Cardoso de Azevedo.

**Methodology:** Whady Hueb, Diogo Freitas Cardoso de Azevedo, Eduardo Gomes Lima.

**Project administration:** Whady Hueb.

**Software:** Cesar Higa Nomura.

**Supervision:** Whady Hueb, Eduardo Gomes Lima, José Antonio Franchini Ramires, Roberto Kalil Filho.

**Validation:** Whady Hueb.

**Visualization:** Paulo Cury Rezende.

**Writing – original draft:** Whady Hueb, Diogo Freitas Cardoso de Azevedo.

**Writing – review & editing:** Diogo Freitas Cardoso de Azevedo.
